# Supplementary figures and images for: Comparative transcriptome analyses of different Salvia miltiorrhiza varieties during the accumulation of tanshinones
Source: PeerJ. 2021 Oct 20;9:e12300. doi: 10.7717/peerj.12300 (PMC8541307; doi:10.7717/peerj.12300)

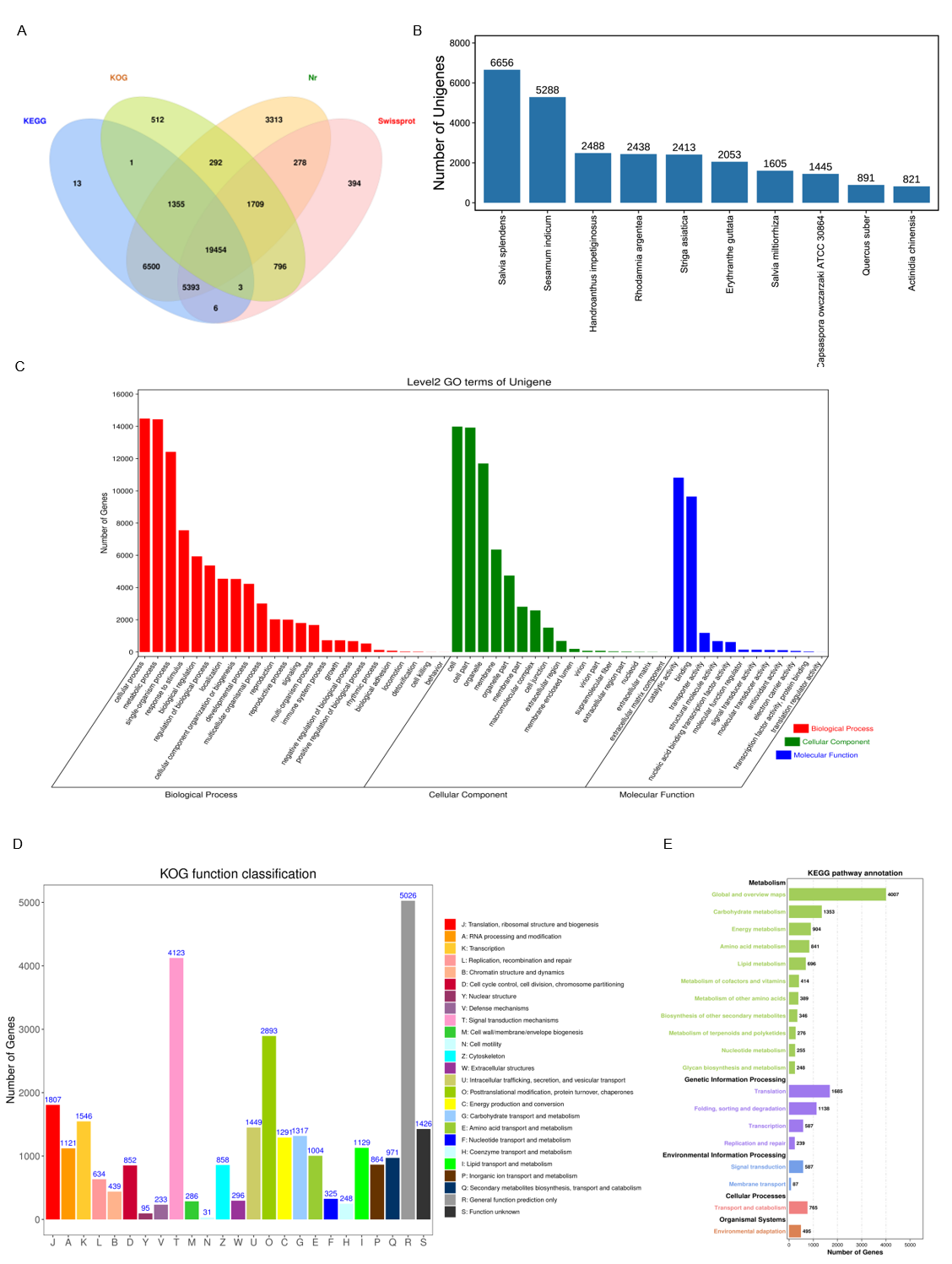

Supplement: Supplemental Information 1 — (A) Venn diagram showing the number of genes annotated in four databases.(B) Species classification against the Nr database for all annotated genes.(C) GO classification of all genes.(D) KOG classification of all genes.(E) KEGG classification of all genes. [file peerj-09-12300-s001.png]

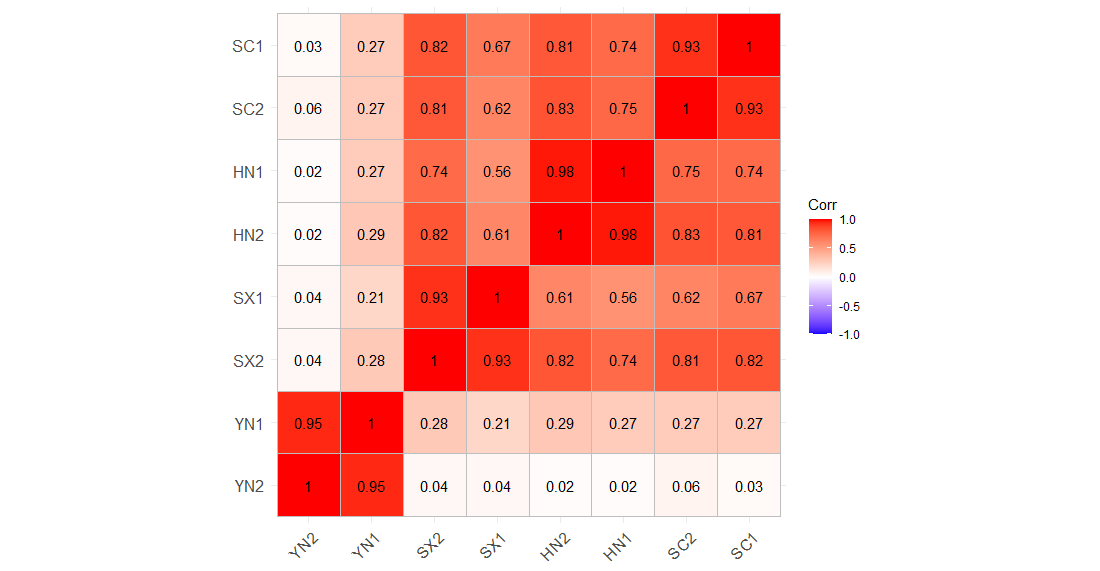

Supplement: Supplemental Information 2 — Abbreviations for the four varieties are as follows: SC (Sichuan), SX (Shanxi), YN (Yunnan) and HN (Henan). The number 1 and 2 after the abbreviations the four varieties represent the early stage of tanshinone accumulation (2 days post-anthesis) and the late stage of tanshinone accumulation (60 days post-anthesis). [file peerj-09-12300-s002.png]

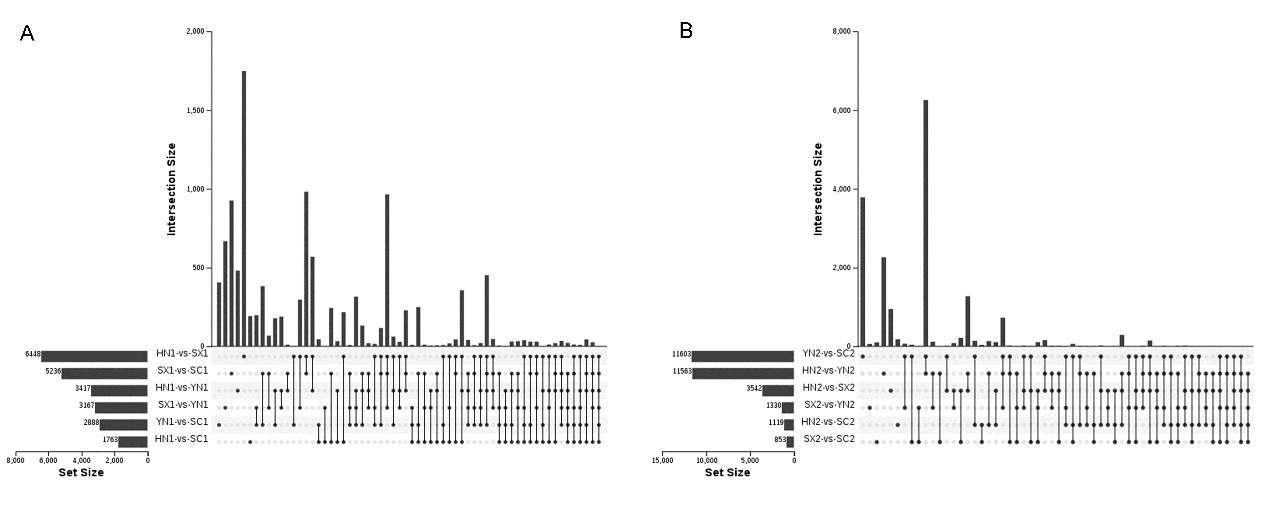

Supplement: Supplemental Information 3 — (A) Differentially expressed genes among the each pairwise comparation groups of four S. miltiorrhiza varieties at stage S1. (B) Differentially expressed genes among the each pairwise comparation groups of four S. miltiorrhiza varieties at stage S2. The horizontal histogram on the left represents the number of differentially expressed genes in each comparison group. The single dot in the middle matrix represents an element unique to the certain comparison group, and the line between the dots represents the unique intersection of different comparison groups. The vertical histograms respectively indicate the number of differentially expressed genes in the corresponding intersection comparison group. Abbreviations for the four varieties are as follows: SC (Sichuan), SX (Shanxi), YN (Yunnan) and HN (Henan). The number 1 and 2 after the abbreviations the four varieties represent the early stage of tanshinone accumulation (2 days post-anthesis) and the late stage of tanshinone accumulation (60 days post-anthesis). [file peerj-09-12300-s003.png]

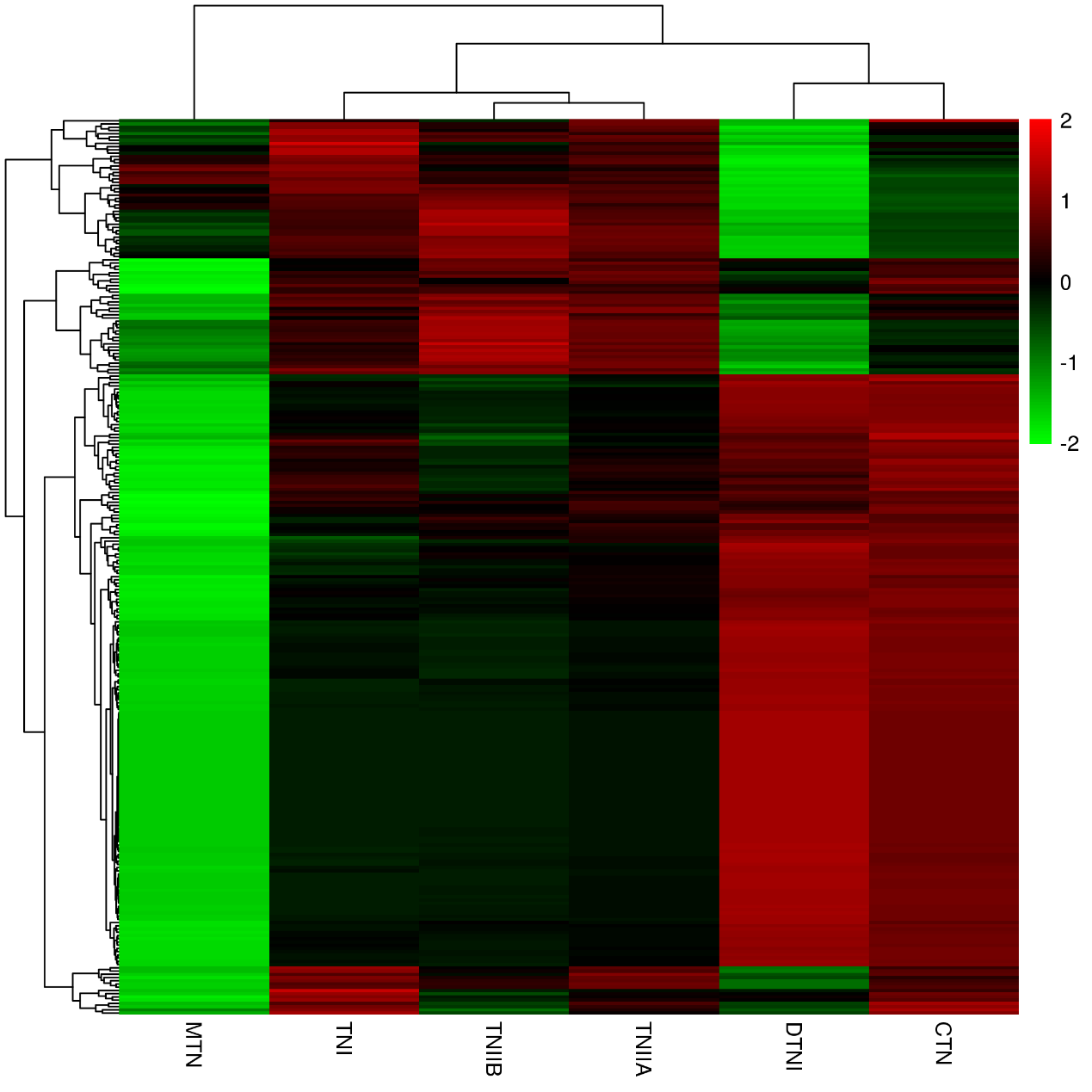

Supplement: Supplemental Information 4 — The X-axis, six tanshinones; Y-axis, 277 tanshinones-related genes. Abbreviations for the six tanshinones are as follows: TNI (tanshinone I), TNIIA (tanshinone IIA), CTN (cryptotanshinone), TNIIB (tanshinone IIB), MTN (miltrinoe), DTNI (dihydrotanshinone I), and TNI (tanshinone I). [file peerj-09-12300-s004.png]

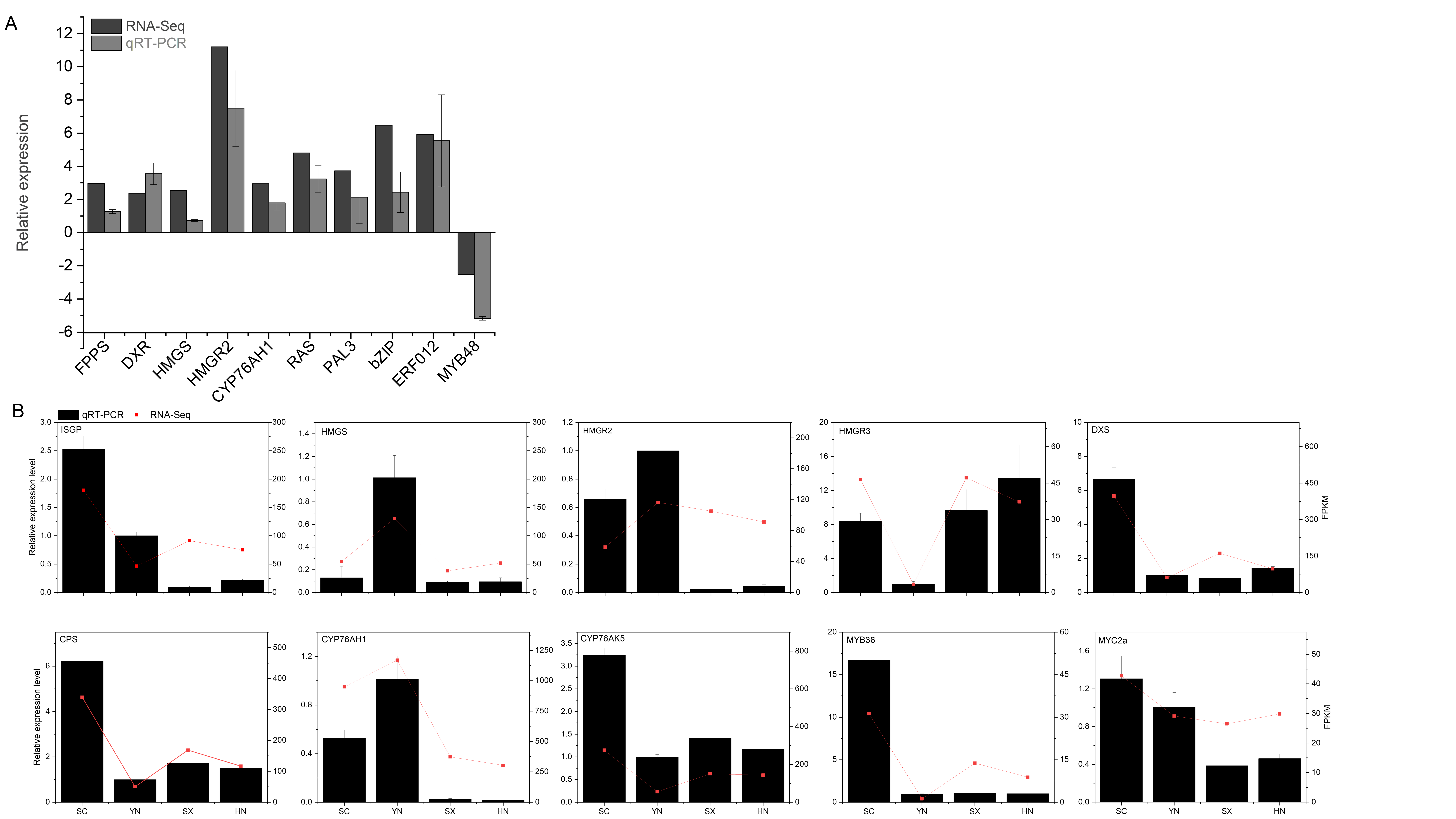

Supplement: Supplemental Information 5 — (A) The qRT-PCR validation of expression profiles obtained by RNA-seq in SC and YN at stage S1. The X-axis represents the 10 selected genes. The Y-axis represents the relative expression levels, Error bars indicate standard error of the mean. (B)The qRT-PCR validation of 10 selected genes in 4 varieties of S. miltiorrhiza at stage S2. Black bars indicate the qRT-PCR results, and red lines show the FPKM values identified via the RNA-Seq analysis. The left Y-axis is the relative expression level of unigenes obtained by qRT-PCR, and the right y-axis denotes the FPKM values in the RNA-Seq data. Error bars indicate standard error of the mean. Abbreviations for the four varieties are as in Fig. S3. Relative expression of these genes was normalized with respect to the β-actin gene. [file peerj-09-12300-s005.png]
